# COVID-19 related reduction in pediatric emergency healthcare utilization – a concerning trend

**DOI:** 10.1186/s12887-020-02303-6

**Published:** 2020-09-07

**Authors:** Christian Dopfer, Martin Wetzke, Anna Zychlinsky Scharff, Frank Mueller, Frank Dressler, Ulrich Baumann, Michael Sasse, Gesine Hansen, Alexandra Jablonka, Christine Happle

**Affiliations:** 1 Department of Pediatric Pneumology, Allergology, and Neonatology, Hannover Medical School, Carl-Neuberg-Straße 1, D - 30625 Hannover, Germany; 2 German Center for Lung Research, Biomedical Research in End Stage and Obstructive Lung Disease, BREATH Hannover, Hanover, Germany; 3 German Center for Infection Research (DZIF), partner site Hannover-Braunschweig, Braunschweig, Germany; 4 Department of Pediatrics, Hannover Medical School, Hannover, Germany; 5 Department of General Practice, University Medical Centre Goettingen, Goettingen, Germany; 6 Department of Pediatric Cardiology and Intensive Care, Hannover Medical School, Hannover, Germany; 7 Pediatric Intensive Care Network Northern Germany/ PIN, Hannover Medical School, Hannover, Germany; 8 Cluster of Excellence RESIST (EXC 2155), Hannover Medical School, Carl-Neuberg-Straße 1, 30625 Hannover, Germany; 9 Department of Rheumatology and Immunology, Hannover Medical School, Hannover, Germany

**Keywords:** SARS-CoV-2, children, pandemic, healthcare utilization, emergency care, COVID-19, pediatric, emergency department

## Abstract

**Background:**

The COVID-19 pandemic has disrupted healthcare systems worldwide. In addition to the direct impact of the virus on patient morbidity and mortality, the effect of lockdown strategies on health and healthcare utilization have become apparent. Little is known on the effect of the pandemic on pediatric and adolescent medicine. We examined the impact of the pandemic on pediatric emergency healthcare utilization.

**Methods:**

We conducted a monocentric, retrospective analysis of *n* = 5,424 pediatric emergency department visits between January 1st and April 19th of 2019 and 2020, and compared healthcare utilization during the pandemic in 2020 to the same period in 2019.

**Results:**

In the four weeks after lockdown in Germany began, we observed a massive drop of 63.8% in pediatric emergency healthcare utilization (mean daily visits 26.8 ± SEM 1.5 in 2019 vs. 9.7 ± SEM 1 in 2020, *p* < 0.005). This drop in cases occurred for both communicable and non-communicable diseases. A larger proportion of patients under one year old (daily mean of 16.6% ±SEM 1.4 in 2019 vs. 23.1% ±SEM 1.7 in 2020, *p* < 0.01) and of cases requiring hospitalisation (mean of 13.9% ±SEM 1.6 in 2019 vs. 26.6% ±SEM 3.3 in 2020, *p* < 0.001) occurred during the pandemic. During the analysed time periods, few intensive care admissions and no fatalities occurred.

**Conclusions:**

Our data illustrate a significant decrease in pediatric emergency department visits during the COVID-19 pandemic. Public outreach is needed to encourage parents and guardians to seek medical attention for pediatric emergencies in spite of the pandemic.

## Background

The novel severe acute respiratory syndrome coronavirus (SARS-CoV-2) causing coronavirus disease 19 (COVID-19) first emerged in Wuhan, China at the end of 2019[1]. Despite efforts to contain the virus, COVID-19 cases emerged all over the world, and the WHO declared a public health emergency of international concern on January 30th. On March 11th 2020, the SARS-CoV-2 outbreak was officially designated a pandemic [2].

Globally, nations responded to this pandemic with lockdown strategies including social distancing, school closures and shelter-in-place orders for non-essential workers. At the same time, healthcare systems in Europe and elsewhere prepared for an unprecedented health emergency by reallocating resources to care for COVID-19 patients [3–6]. Public awareness of the pandemic was associated with a vast reduction in healthcare utilization in Italy and other countries [7–10]. As a result, increased adult and pediatric morbidity and mortality due to delayed healthcare utilization were reported [11, 12].

Initially, Germany took a moderate approach, appealing to the responsibility and solidarity of its citizens. However, school closures were implemented beginning on March 16th, and an official lockdown of public life, including mandatory closure of all non-essential retail businesses, was implemented starting on March 23rd 2020. Severe cases of COVID-19 remained comparatively low in Germany (n = 163,175 confirmed SARS-CoV-2 infections with 6.692 fatalities as of May 4th 2020 [13]), and the healthcare system has thus far been spared an untenable influx of critically ill patients.

In this setting, we investigated pediatric emergency healthcare utilization in our tertiary care center in Hanover, Germany. We compare the rate and type of emergency department (ED) visits during lockdown in March and April of 2020 to the equivalent period in the spring of 2019.

## Methods

### Study population

We analysed data from n = 05,424 healthcare visits occurring between January 1st and April 19th 2019 and January 1st and April 19th 2020 in the pediatric ED of Hanover Medical School in Hanover, Lower Saxony. We also compared healthcare utilization between calendar weeks 12–15 (March 18th to April 14th ) in 2019 and calendar weeks 12–15 (March 16th to April 12th ) in 2020. The ED is open to patients 24 h a day, seven days a week, with full-time coverage by a designated nurse and pediatric physician. During daytime hours, from 8 am to 5 pm Monday through Thursday and 8 am to 4:30 pm on Fridays, a designated pediatric ED physician provides care. Patients presenting to the ED outside these times are seen by pediatric house staff and/or an on-call pediatric hospitalist.

### Data collection

All medical and demographic data were extracted from routine electronic clinical records. Every onsite healthcare visit was documented in an electronical case reporting system, including the patient’s age and sex, date and time of visit, complaints, and suspected or confirmed diagnoses, as well as whether hospitalization was required. In our hospital, standard practice dictates that the diagnosis or primary complaint of each ED patient is recorded during the visit. Diagnoses are coded according to the International Statistical Classification of Diseases and Related Health Problems system (ICD)-10-GM-2020 by trained coding personnel. Coding personnel receive intensive training in ICD coding, and is specialized and experienced in this field. Coding is continuously supervised, monitored, and reviewed for interrater agreement. Interdisciplinary meetings between medical and coding personnel to discuss unclear cases are held regularly. As analyses for this paper were retrospective, coding was blinded to the study objectives. All consultations between March 18th (0:00 AM) and April 14th 2019 (12:00 PM) and from March 16th (0:00 AM) to April 12th (12:00 PM) were included; no cases were excluded. All data was extracted in depersonalized form. ICD diagnoses were grouped into categories that were defined as follows: the category “communicable diseases” was defined as either diagnoses classified in group “A/B” of the (ICD)-10-GM-2020 coding system (certain infectious and parasitic diseases) or as organ system-specific reporting of an infectious disease (e.g. for airway infections, diagnoses such as cold (J00) or unspecified type of upper airway infection (J06.9) were included). For analyses of suspected infectious diseases, the coding of the following signs and symptoms were included: fever, cough, stridor, dyspnoea, vomiting, and diarrhoea. All other diagnoses or complaints were defined as “noncommunicable diseases”. For the illustrations in Figs. 3 and 4, patients with signs, symptoms, and findings from ICD-10 groups with low prevalence or from the category “R” (symptoms, signs and abnormal clinical and laboratory findings, not elsewhere classified), or those with unknown complaints were grouped into the category “others”.

### Statistical analyses

SPSS version 24.0 and Graphpad Prism version 5.02 were used. Normal distribution of all variables was assessed, and depending on data distribution, group differences were evaluated by Student´s T or Mann Whitney U testing. P values below 0.05 were considered significant.

### Ethics compliance

The analyses were approved by the Data Security Management and Institutional Review Board of Hannover Medical School.

## Results

**Fig. 1:**
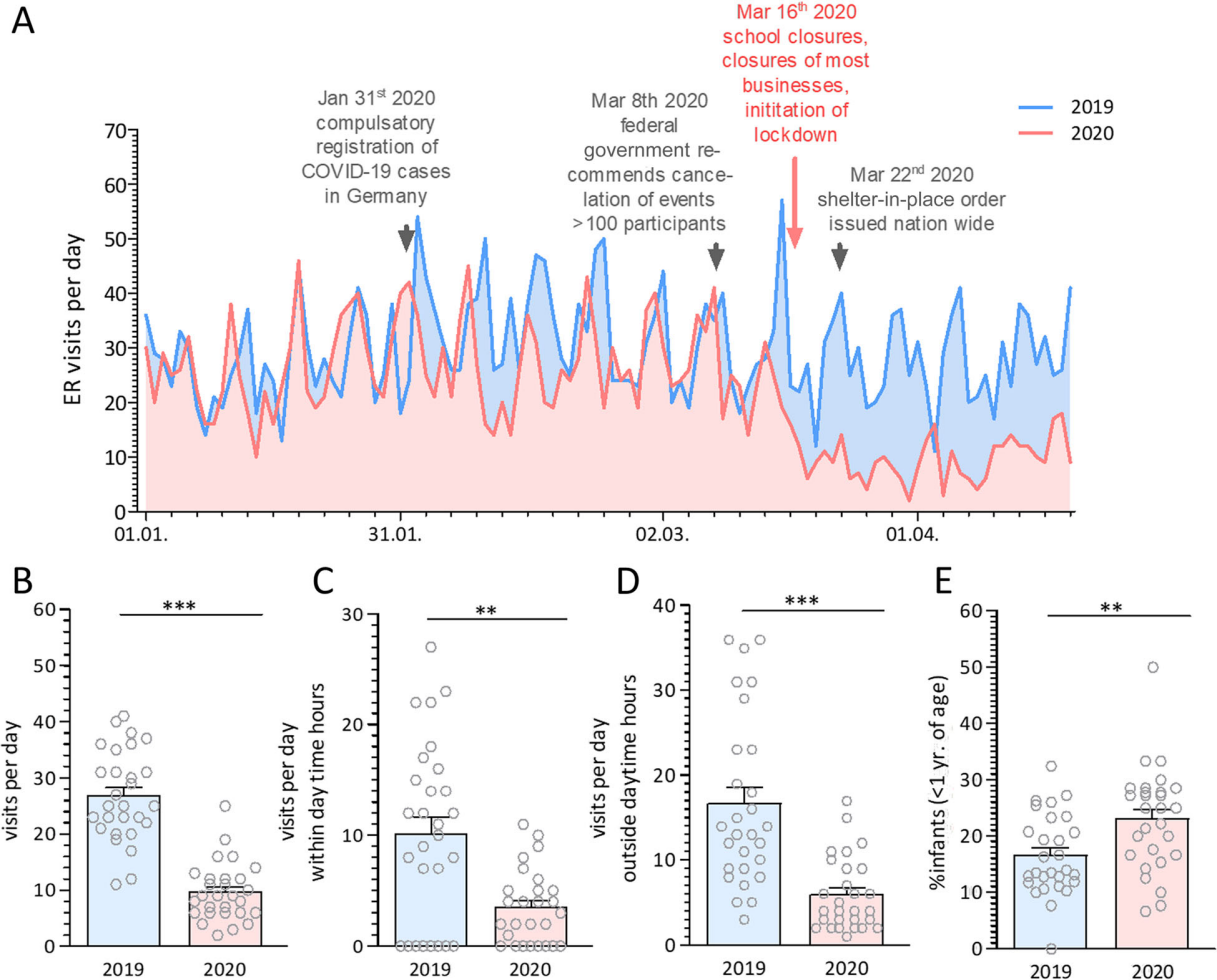
Reduced pediatric emergency healthcare utilization after the implementation of pandemic-related lockdown in Germany: A: daily number of visits between January 1st and April 19th 2019 (blue) vs. 2020 (red) including dates of COVID-19 related policy in Germany. B: visits per day in calendar weeks 12 to 15, C/D: visits per day within (C) and outside (D) ED daytime service in calendar weeks 12 to 15. E: increased proportion of daily visits of patients younger than one year (bars display mean + SEM (B-E) with overlaying dots representing single daily values, ** *p* ≤ 0.01, *** *p* ≤ 0.001)

In total, *n* =5,424 visits occurred during the analysed period between January 1st and April 19th 2019 and January 1st and April 19th of 2020 in the pediatric ED of our hospital. The mean age of all patients included in this analysis was 7.1 years (± SEM 0.1 yrs.), and 49.5% of cases were female. Given that primary pediatric care in Germany is usually provided by pediatric practices during weekdays and lockdown did not include closure of pediatric practices, in both observational periods more visits to the ED occurred during weekends. Overall, a mean of 16.4 ± SEM 1.5 visits per day on Saturday/Sunday as compared to 13.5 ± SEM 0.4 per weekday occurred. When we compared daily visit frequencies between 2019 and 2020, we observed a striking reduction in the number of patients presenting to the ED after March 16th 2020, the day that school closures and a nationwide lockdown began (Fig. 1a).

In calendar weeks 12 to 15 of 2020, case numbers decreased by 63.8% compared to the same time period in 2019 (Table 1; Fig. 1b). This reduction in emergency healthcare utilization was observed during daytime as well as overnight (Table 1; Fig. 1c, d).

**Table 1:** Comparison of healthcare utilization, specific diagnoses and complaints and hospitalisation after the implementation of pandemic-related lockdown in Germany

item	daily mean (±SEM)		*p*-value
	2019	2020	
overall ED visits (*n*=)	26.8 (1.5)	9.7 (1)	0.005
visits during daytime hours (*n*=)	10.1 (1.5)	3.5 (0.6)	< 0.001
visits outside daytime hours (*n*=)	16.6 (1.9)	5.9 (0.8)	< 0.001
patient age (years)	5.8 (0.2)	5.8 (0.3)	p0.64
ED visits by children < 1year of age (%)	16.6 (1.4)	23.1 (1.7)	< 0.01
ED visits by children < 6yrs. of age (%)	62 (1.9)	61.7 (2.9)	p0.93
proportion of female patients (%)	44.9 (1.6)	42.5 (2.7)	p0.39
patients with noncommunicable diseases (*n*=)	11.2 (0.7)	4.7 (0.5)	< 0.001
patients with communicable diseases (*n*=)	15.1 (1.5)	4.5 (0.5)	< 0.001
patients with communicable diseases (%)	45 (2.9)	54.5 (3.9)	p0.19
patients with intoxications or injuries (*n*=)	1.3 (0.2)	0.3 (0.1)	*p* < 0.001
patients with diagnoses affecting the GIT (*n*=)	0.1 (0.2)	0.4 (0.1)	*p* < 0.001
patients with diagnoses affecting the eyes or ears (*n*=)	0.7 (0.2)	0.1 (0.1)	*p* < 0.001
patients with respiratory infections or signs thereof (*n*=)	4.9 (0.5)	1.6 (0.3)	*p* < 0.001
patients with ear and throat infections or signs thereof (*n*=)	2.5 (0.4)	0.3 (0.1)	*p* < 0.001
patients with GIT infections or signs thereof (*n*=)	2.9 (0.5)	0.4 (0.1)	*p* < 0.001
patients hospitalised from the ED (*n*=)	4.9 (0.5)	3 0.3)	*p* < 0.001
hospitalisation rate of all ED patients (%)	13.9 (1.6)	26.6 (3.3)	*p* < 0.001
duration hospitalisations of hospitalised ED patients (days)	5.5 (0.6)	4.5 (0.3)	p 0.9

While the mean age of patients did not differ significantly between calendar weeks 12 to 15 in each year, a significant increase in ED visits by infants below the age of one year occurred in between 2019 and 2020 (Table 1; Fig. 1e). Although most shifts in daily healthcare utilization were small in absolute numbers (e.g. a mean of one less intoxication case per day in 2020 vs. 2019), most differences were highly significant when examining the entire observational period (Table 1). No significant differences in the daily proportion of school children below the age of six years between the two analysed periods was observed, nor did we find a difference in sex distribution (Table 1).

**Fig. 2:**
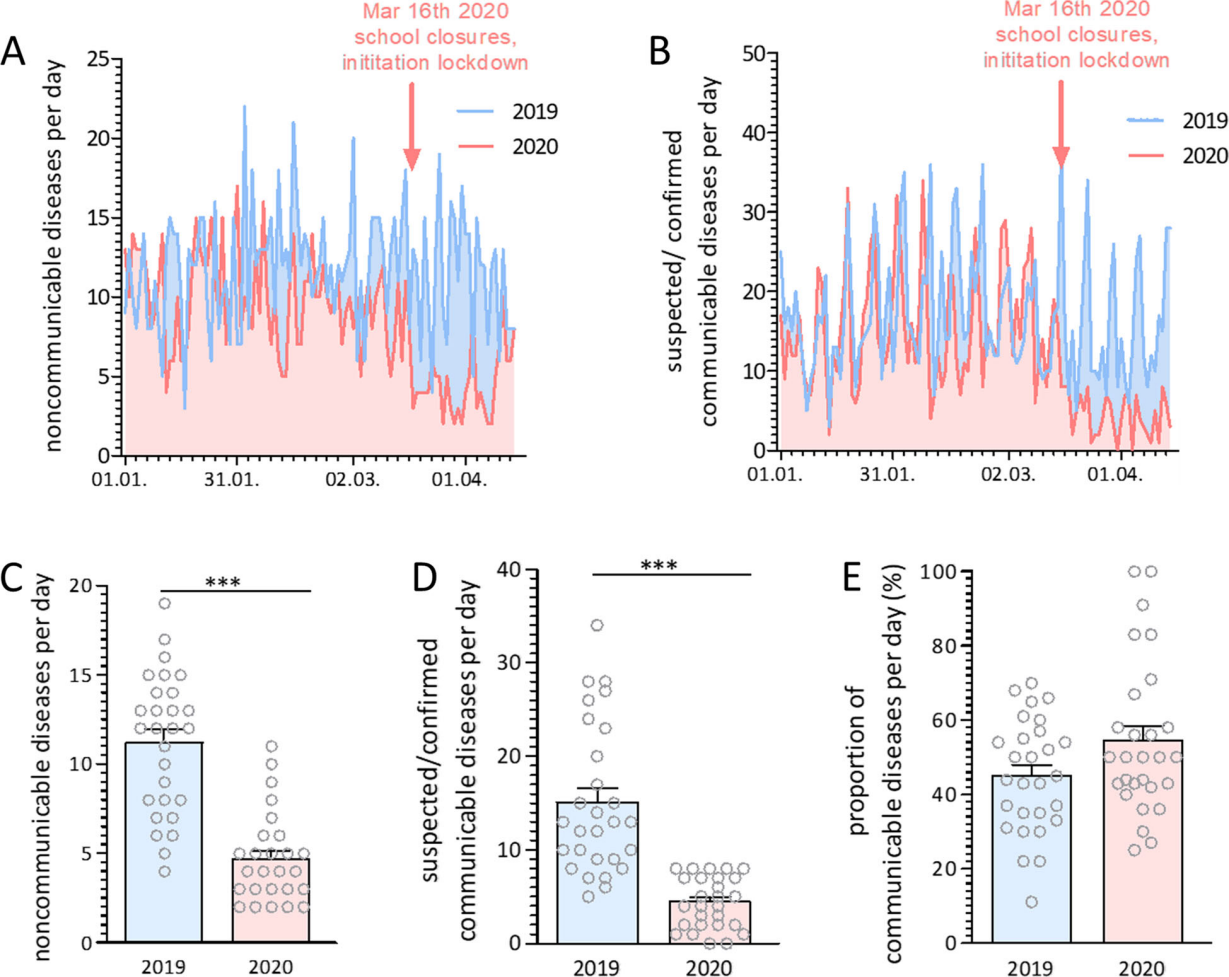
ED cases of patients presenting with noncommunicable disease as well as those with infectious disease or signs thereof were significantly reduced during the COVID-19 pandemic. A: daily number of visits between January 1st and April 19th 2019 (blue) vs. 2020 (red) by patients with noncommunicable diseases (A) and infectious diseases or signs thereof (B). C/D: reduced visits per day due to noncommunicable (C) or infectious diseases or typical signs thereof in calendar weeks 12 to 15. E: no change in the proportion of daily visits due to infectious diseases or typical signs thereof (bars display mean + SEM with overlaying dots representing single daily values (C-E), *** *p* ≤ 0.001)

Next, we analysed the complaints or diagnoses recorded in the ED in both years. Diagnoses of primary complaints were reported in n = 5,206 visits (96%). A clear drop in the frequency of daily visits for noncommunicable diseases and for confirmed infections or signs thereof (Fig. 2a/b) was observed.

This was confirmed by a separate analysis comparing the frequency of daily visits for noncommunicable diseases in calendar weeks 12 to 15 of 2019 to the same period in 2020. Here, a significant decrease (58%) (Table 1; Fig. 2c) was observed.

Daily visits for suspected or confirmed communicable diseases displayed a significant decrease (70.2%) in the four weeks after lockdown began (Table 1; Fig. 2c). The proportion of daily patients with infectious diseases or signs and symptoms of infection, however, did not differ significantly between calendar weeks 12 to 15 from one year to another (Table 1; Fig. 2e).

**Fig. 3:**
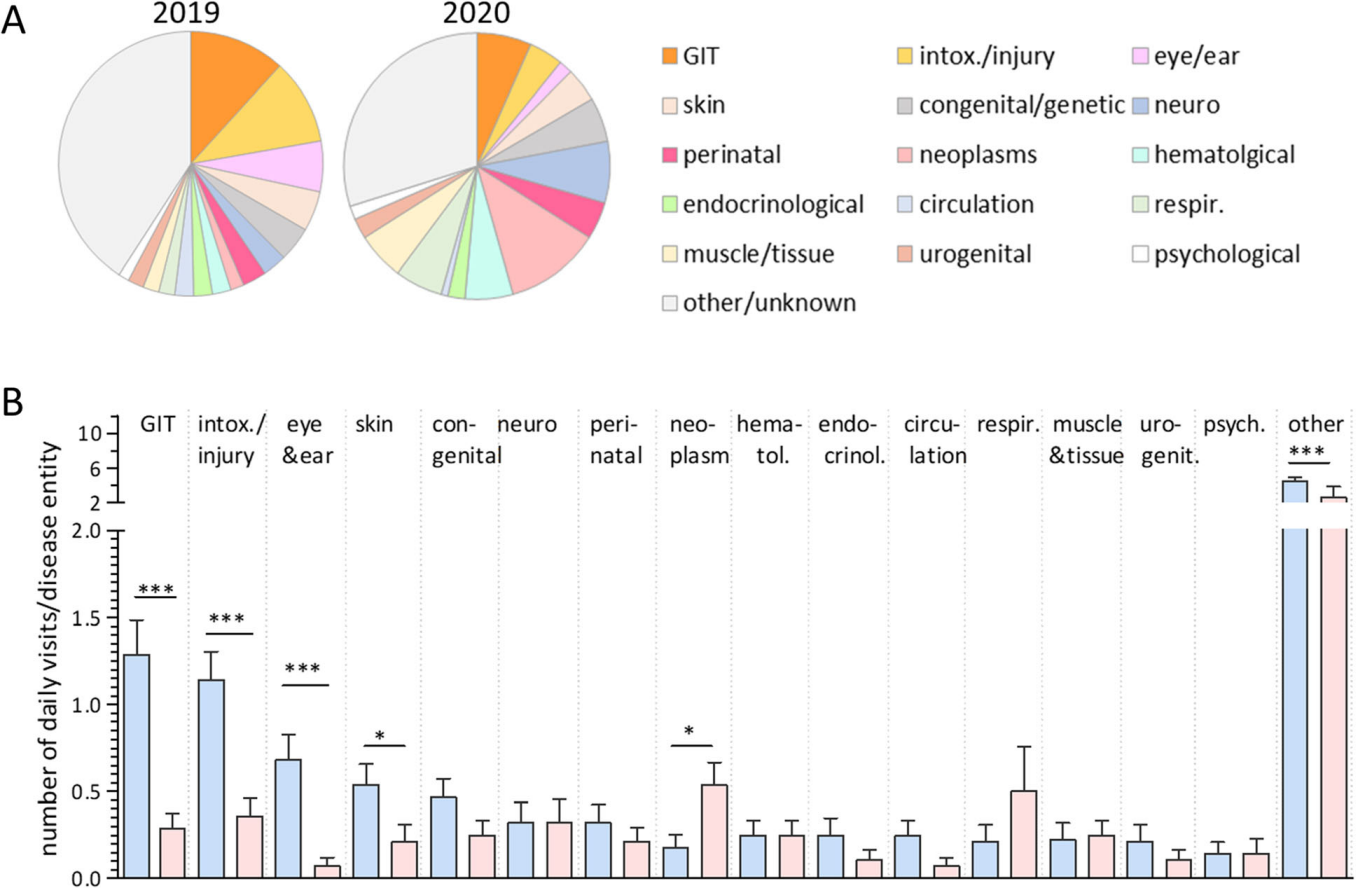
Distribution and frequency of ED presentation due to noncommunicable disease: A: proportion of organ system specific diagnoses in calendar weeks 12 to 15. B: daily number of visits per organ system specific disease entity in calendar weeks 12 to 15 (GIT: diseases/complaints of the gastrointestinal tract, intox.: intoxication, neuro: neurological diseases/complaints, neoplasm: neoplastic/malignant diseases/complaints, hematol.: hematological diseases/complaints, endocrinol.: endocrinological diseases/complaints, respir.: respiratory diseases/complaints, urogen.: urogenital diseases/complaints, psych.: psychiatric diseases/complaints ; bars display mean + SEM, * *p* ≤ 0.05, ** *p* ≤ 0.01, *** 0.001)

We also investigated the specific complaints leading to pediatric ED visits in the calendar weeks 12 to 15 in 2019 and 2020. In both years, a proportion of diagnoses was either unknown or unspecific (in total 45.6% of diagnoses in 2019 and 32.9% of diagnoses in 2020). For reported diagnoses, the pattern of distribution was similar in both years. This held true even for critically ill patients. For example, during the four week period we analysed, we observed three seizures in 2019, and two seizures in 2020. As shown in Fig. 3a, however, some variation was observed, such as a larger proportion of patients presenting with malignant or neoplastic diseases in 2020 (1.6% 2019 vs. 11.6% in 2020). This change was confirmed by comparing absolute patients numbers (Fig. 3b). The only disease category with increased daily ER visits after the lockdown began was that of malignant/neoplastic disease (Table 1; Fig. 3). For the vast majority of noncommunicable diseases, daily visits were reduced (Fig. 2b). For example, complaints or diagnoses affecting the gastrointestinal system showed a significant decrease of 77.8%, cases of intoxication or injury showed a significant 68.8% reduction, and diagnoses and complaints affecting the eyes or ear were reduced significantly by 89.5% in 2020 compared to 2019 (Table 1; Fig. 3).

**Fig. 4:**
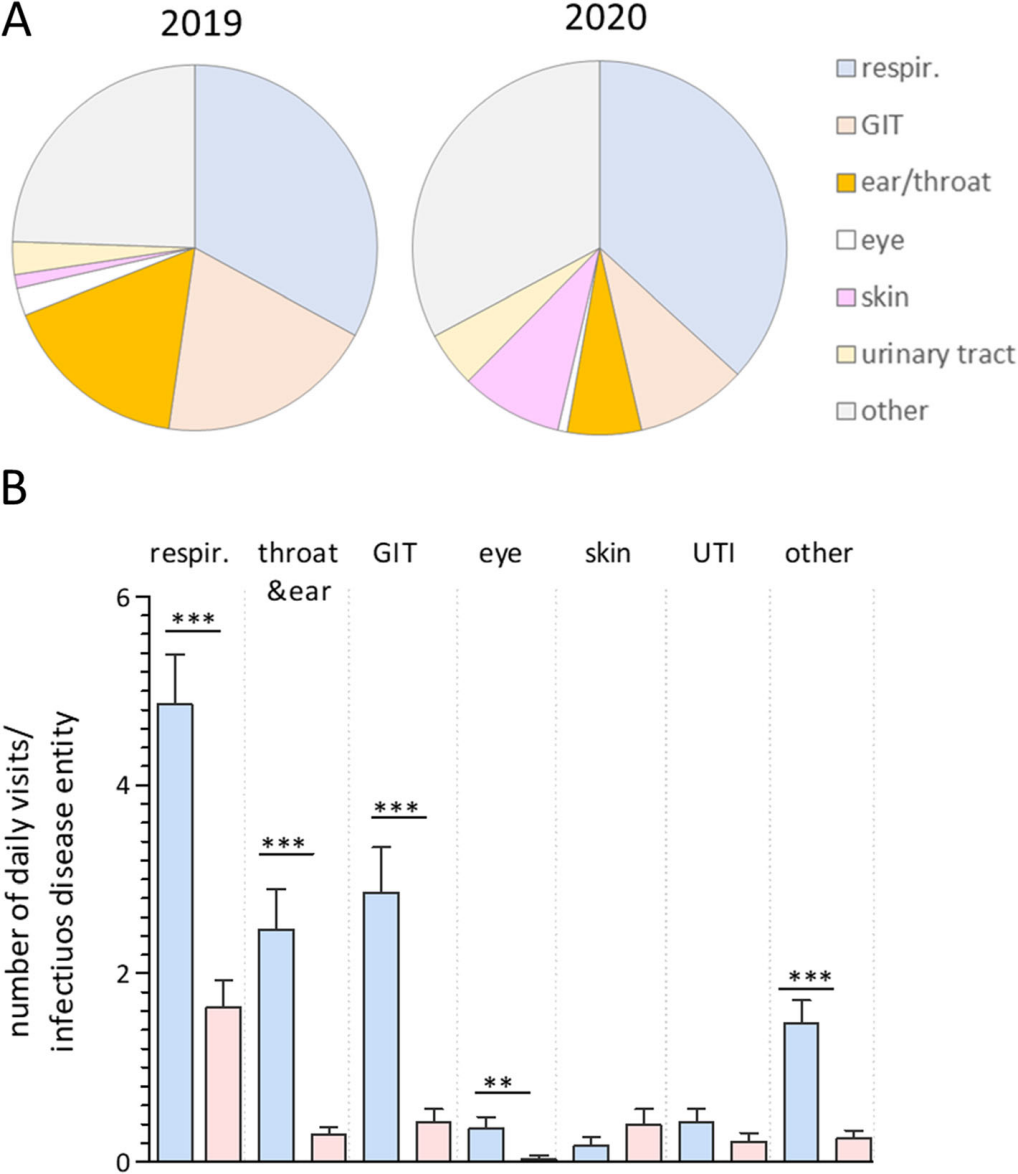
Distribution and frequency of infectious disease after lockdown began: A: proportion of organ system specific diagnoses in calendar weeks 12 to 15. B: daily number of visits per organ system specific disease entity in calendar weeks 12 to 15 (respir.: respiratory infections, GIT: infections of the gastrointestinal tract, UTI.: urinary tract infections; bars display mean + SEM, ** *p* ≤ 0.01, *** 0.001)

Similarly, the proportion of organ-specific infectious diseases or signs thereof was analysed for the two time periods. As shown in Fig. 4a, the majority of daily diagnosed infections or typical complaints thereof concerned the respiratory or gastrointestinal tracts. In absolute numbers, presentations due to organ-specific infectious disease decreased significantly during calendar weeks 12 to 15 of 2020 as compared to the same period in 2019 (Table 1; Fig. 4b). Respiratory infections or signs thereof were reduced significantly by 89%, ear and throat infections or signs showed a significant reduction of 88.2%, and gastrointestinal infections or signs thereof were also reduced significantly by 85% (Table 1; Fig. 4b).

**Fig. 5:**
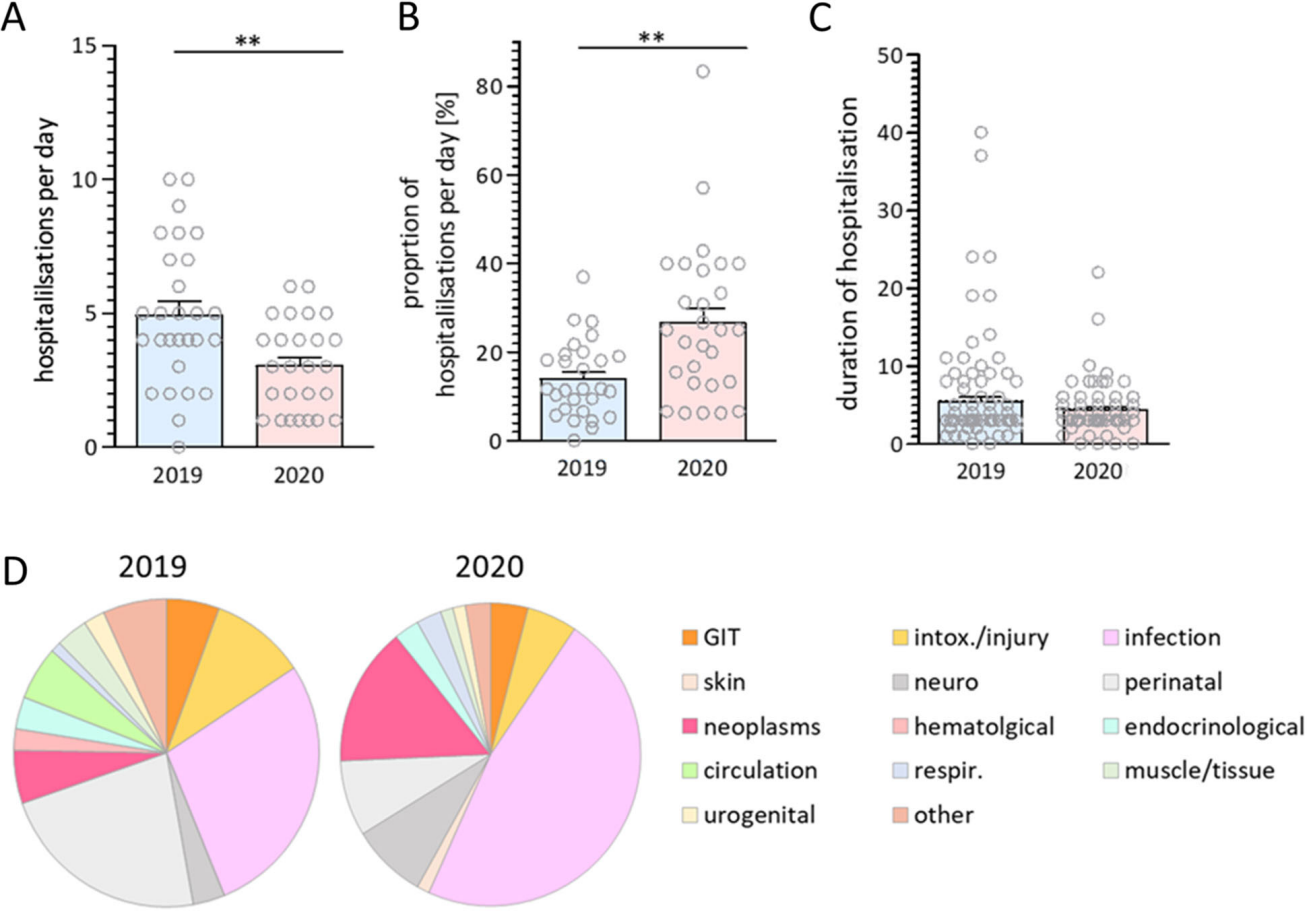
Frequency of hospital admission from our pediatric ED during pandemic-related lockdown: A: reduced numbers of daily hospitalisations in calendar weeks 12–15 in 2019 vs. 2020. B: increase in proportions of hospitalised patients per ED visit during the same periods, C: no difference in the duration of hospital stay D: distribution of diagnosis groups in hospitalised patients in 2019 vs. 2020 (GIT: diseases/complaints of the gastrointestinal tract, intox.: intoxication, respir.: respiratory diseases/complaints, bars display mean + SEM, ** *p* ≤ 0.01)

Finally, we analysed the rates of hospitalisation in the first month after pandemic-related school closures in 2020 as compared to the same period of time the previous year. Although the absolute numbers of daily hospitalisations of pediatric ED patients dropped significantly (38.4% decrease; Table 1; Fig. 5a), the proportion of hospitalisations among all patients presenting to the ED almost doubled in 2020 compared to 2019 (Table 1; Fig. 5b). The duration of hospitalisation, however, was not different, neither overall (Table 1; Fig. 5c) nor for particular subgroups such as patients below the age of one year, those with infectious diseases, or those requiring intensive care.

We observed a similar pattern of diagnoses leading to hospitalisation from the ED in both years. Infectious disease, perinatal pathologies, injuries, gastrointestinal issues and neoplastic disease belonged to the most common indications for admission. The largest changes in absolute patient numbers were observed in patients with neoplastic diseases (5.6% in 2019 vs. 14.9% in 2020), those with pathologies of the perinatal period (22.5% in 2019 vs. 8.1% in 2020) and those with diseases of the heart and circulatory system (5.6% in 2019 vs. 0% in 2020).

During calendar weeks 12 to 15 in 2019, two patients presenting to the ED were admitted to our hospital´s pediatric intensive care unit (PICU). No fatalities occurred amongst these patients. In the same four-week period in 2020, two patients were admitted from the ED directly to the PICU, and no deaths were observed. Only one case of pediatric COVID-19 was diagnosed during the analysed period.

## Discussion

Our data demonstrate a significant decrease in pediatric ED visits during the COVID-19 lockdown in Germany. This finding is consistent with other reports on the substantial reduction in healthcare utilization in Europe and elsewhere during the current crisis [9, 10]. Although a pandemic-related reduction in primary healthcare utilization for critically ill patients has been reported [7, 8, 11], little is known on the changes in pediatric emergency medicine thus far.

Lazzerini et al. recently reported twelve pediatric cases of delayed treatment due to parental concern of nosocomial COVID-19 infection from a healthcare facility [12]. Tragically, half the patients in this study were admitted to a PICU and four of them died.

We do not know whether the reduction in healthcare utilization in our ED in 2020 occurred due to lower morbidity or avoidance of hospital visits associated with the current pandemic. Although we can only speculate on the causes behind this dramatic reduction in case numbers, the following reasons appear to be most relevant.

Firstly, a massive flow of alarming medical information has resulted in high levels of uncertainty and fear across Europe and elsewhere [14]. Although children are less prone to severe courses of COVID-19 [15], the fear of contracting SARS-CoV-2 while visiting a doctor´s office or hospital may have affected parental decision-making during the early phase of this unprecedented health emergency [12]. In this context, effective public health communication is a key factor, not only in fighting the spread of COVID-19, but in preventing healthcare avoidance for those in need [12, 16]. Currently, limited scientific understanding of the virus and its spread impedes evidence-based guidance for the public, which poses a significant challenge to health communicators worldwide [2]. However, pediatricians should emphasize to parents and young patients that the risks of avoiding hospital care in emergencies may exceed that of contracting SARS-CoV-2. The fear of infection may not be entirely irrational in times of reported patient overflow, overcrowded waiting areas, and insufficient personal protective equipment for medical staff [16, 17]. But in Germany, where the healthcare system thus far has not been overwhelmed by COVID-19, the risk of nosocomial infection within a hospital setting appears, at least for now, to be low [18]. During the period of time analysed in this study, in spite of extensive testing, only one child tested positive for SARS-CoV-2 in our pediatric emergency department.

The second likely reason for the reduction in ED visits, in particular for infectious diseases, lies in the lockdown strategies themselves. Even before governmental enforcement, many families practised rigorous social distancing. This measure, together with school closures, cancelation of public gatherings and shelter-in-place orders, led to a dramatic decrease in the spread of communicable disease. For example, the influenza season in Germany came to a sudden and almost complete halt by calendar week 14 of 2020 [19]. This may also affect overall morbidity and mortality in children. While the publication from Italy reporting on pediatric deaths associated with parental fear of seeking emergency healthcare is alarming [12], overall pediatric mortality in Europe appears have dropped during the COVID-19 pandemic [20].

Another possibility is that, out of respect for the limited resources of the medical workforce during the pandemic, parents may have been inclined to “watch and wait” when their children fell ill, as opposed to immediately presenting to the ED. The increased proportion of infants and hospital admissions from the ED in calendar weeks 12 to 15 of 2020 suggests that children at particular risk for severe disease were brought to the ED despite the pandemic, but that those with non-severe illnesses were presented less frequently. Fortunately, no fatalities were observed in our analysis. Although an increased rate of daily hospital admissions occurred, we found comparable durations of hospital stay and numbers of ICU admission between 2019 and 2020. This supports the idea that, despite the pandemic, critically ill patients presented to our ED in time.

The overall low case numbers of severely ill children must to be taken into account when interpreting our data. Although significant, many shifts in daily emergency care utilization such as the reduction in cases of intoxication or diagnoses affecting the digestive system were small in absolute numbers and should not be overinterpreted. The incidence of PICU admissions, as well as of specific severe disease types such as seizures or neonatal sepsis, were low in both time periods studied, prohibiting far-reaching conclusions. The Northern German Pediatric Intensive Care Network “PIN”, which is headquartered in Hanover and serves as a reference centre for more than 45 pediatric hospitals in Northern Germany, also reported no change in the number or severity of PICU admissions in calendar week 12–15 in 2020 as compared to 2019. Another limitation of our work lies in the fact that, due to the nature of our ED documentation system, in many instances only the primary complaint was recorded, rather than a concrete diagnosis. Emergency medicine chart reviews, by nature, are flawed, because the underlying data was not collected for scientific purposes [21]. Furthermore, only the acute reason for presentation was analysed, which does not account for the many patients at our university hospital who suffer from complex chronic conditions. We do not know why more children with neoplastic diseases presented to the ED after lockdown as compared to the same period in time in 2019; and further analyses are needed to address healthcare utilization and complaints in specific subgroups such as chronically ill children during the current crisis.

Thus far, Germany has taken a moderate approach to lockdown strategies with a combination of legal restrictions to shut down public life and an appeal to the responsibility and solidarity of its citizens. The healthcare system, particularly in Northern Germany, was in the fortunate situation of having weeks to adapt and prepare for this unprecedented situation and has been able to provide adequate care to COVID-19 patients [22, 23]. In spite of the low prevalence of COVID-19 in our region, our data shows the profound impact of the pandemic on pediatric emergency health care utilization.

## Conclusions

The current pandemic puts pressure on healthcare systems around the globe. Our data clearly indicate that the COVID-19 outbreak is associated with a significant reduction in pediatric emergency healthcare utilization. Although we did not observe any critical cases due to delayed hospital admission in our analysis, the risk of avoidance or delay of healthcare provision to critically ill children and adolescents may only become apparent in the months and years to come. Germany is still in the fortunate position of having sufficient resources for patient care, but our data suggest that the current pandemic results in a dramatic reduction in the use of emergency medical care for children in our system, presumably based on fear of hospital acquired infections or out of respect for the limited resources of the healthcare system. This may affect resource-limited healthcare systems that are struck by COVID-19 to greater extent - as the fear of infection or the ensuing chaos could be more pronounced in these regions. It is important to note that the phenomenon of reduced pediatric healthcare utilization may extend beyond emergency medicine to insufficient healthcare provision and preventive medicine for children in general. As such, public health communication should aim at minimizing possible pediatric “collateral damage” caused by delayed emergency healthcare utilization during the COVID-19 pandemic. We hope our analysis helps to adapt healthcare and communication strategies accordingly.

## Data Availability

The dataset necessary to interpret, replicate and build upon the findings reported in the article will be made available on reasonable request and can be obtained by contacting the corresponding author.

## Abbreviations

COVID-19: Coronavirus disease 19
ED: Emergency department
ICD-10: International Statistical Classification of Diseases and Related Health Problems 10
SARS-CoV-2: Severe acute respiratory syndrome coronavirus 2
SEM: Standard error mean
WHO: World health organisation
